# T cell repertoire breadth is associated with the number of acute respiratory infections in the LoewenKIDS birth cohort

**DOI:** 10.1038/s41598-023-36144-x

**Published:** 2023-06-12

**Authors:** Lisa Paschold, Cornelia Gottschick, Susan Langer, Bianca Klee, Sophie Diexer, Ivona Aksentijevich, Christoph Schultheiß, Oliver Purschke, Peggy Riese, Stephanie Trittel, Roland Haase, Frank Dressler, Wolfgang Eberl, Johannes Hübner, Till Strowig, Carlos A. Guzman, Rafael Mikolajczyk, Mascha Binder

**Affiliations:** 1 Department of Internal Medicine IV, Oncology/Hematology, Martin-Luther-University Halle-Wittenberg, Ernst-Grube-Str. 40, 06120 Halle (Saale), Germany; 2 Interdisciplinary Center for Health Sciences, Institute for Medical Epidemiology, Biometrics and Informatics (IMEBI), Medical School of the Martin-Luther University Halle-Wittenberg, Magdeburger Strasse 8, 06112 Halle (Saale), Germany; 3 Inflammatory Disease Section, National Human Genome Research Institute, National Institutes of Health, Bethesda, MD USA; 4 Department Vaccinology and Applied Microbiology, Helmholtz Centre for Infection Research, 38124 Braunschweig, Germany; 5 Department of Neonatology and Pediatric Intensive Care, Hospital St. Elisabeth und St. Barbara, 06110 Halle (Saale), Germany; 6 Department of Pediatric Pulmonology, Allergology and Neonatology, Hannover Medical School, 30625 Hannover, Germany; 7 Department of Paediatrics, Hospital Braunschweig, 38118 Braunschweig, Germany; 8 Department of Paediatrics, Dr. von Hauner Children’s Hospital, Ludwig- Maximilians-University Munich, 80337 Munich, Germany; 9 Department Microbial Immune Regulation, Helmholtz Centre for Infection Research, 38124 Braunschweig, Germany; 10 Division of Medical Oncology, University Hospital Basel, Petersgraben 4, 40314031 Basel, Switzerland

**Keywords:** Immunogenetics, Viral infection, Risk factors, Immunology, Immunogenetics, Infection, Lymphocytes

## Abstract

We set out to gain insight into peripheral blood B and T cell repertoires from 120 infants of the LoewenKIDS birth cohort to investigate potential determinants of early life respiratory infections. Low antigen-dependent somatic hypermutation of B cell repertoires, as well as low T and B cell repertoire clonality, high diversity, and high richness especially in public T cell clonotypes reflected the immunological naivety at 12 months of age when high thymic and bone marrow output are associated with relatively few prior antigen encounters. Infants with inadequately low T cell repertoire diversity or high clonality showed higher numbers of acute respiratory infections over the first 4 years of life. No correlation of T or B cell repertoire metrics with other parameters such as sex, birth mode, older siblings, pets, the onset of daycare, or duration of breast feeding was noted. Together, this study supports that—regardless of T cell functionality—the breadth of the T cell repertoire is associated with the number of acute respiratory infections in the first 4 years of life. Moreover, this study provides a valuable resource of millions of T and B cell receptor sequences from infants with available metadata for researchers in the field.

## Introduction

Newborns, infants and young children are more susceptible to infection than adults^1,2^. The spectrum ranges from sporadic mild diseases such as seasonal acute respiratory infections to severe infections in the immediate postnatal period^3^. Different factors may impact the frequency and severity of early life infections: the degree of immune immaturity and integrity of barrier defenses (e.g. in preterm infants), exposure to pathogens through early social interactions (e.g. daycare or having siblings), as well as genetic traits ranging from polygenic predisposition to primary immunodeficiencies through monogenetic inborn errors of the immune system. Children with primary immunodeficiencies often show unusual infection manifestation, but account only for a small minority of cases^4,5^. For most children with increased vulnerability to infection and without evident genetic or environmental triggers, we lack insight into the immune configurations that may underlie these phenotypes.

While the innate branch of the immune system provides a first and rather unspecific line of defense against pathogens, B and T cells of the adaptive immune system act in a more sophisticated way to control infections by recognizing epitopes with their unique antigen receptor formed through genetic recombination of V, D and J genes. In young children, adaptive immunity is not deficient as evidenced by specific responses even to fetal infections^6^, but a certain degree of immaturity is evident in early life. Both the fetal T and B cell repertoire begin to form and diversify as early as at the end of the first trimester of pregnancy^7,8^. Thereby, T cells still retain a particular epigenetic program and a more rapid onset of exhaustion in the newborn^9^ and B cell responses to some vaccines increase with age at immunization^10^. High throughput adaptive immune receptor repertoire sequencing (AIRR-seq) of B and T cells has opened up avenues for the in-depth characterization of immune architectures in various tissues as well as—most prominently—in the peripheral blood. The shapes of such receptor repertoires are based on the antigens encountered throughout life, therefore repertoire “snapshots” provide information on the current immune status as well as the antigen history. Many investigations have shown the effects of aging and imprints of autoimmunity, specific infections, or vaccinations on the receptor repertoire over a lifetime^11–17^. Most of these studies have been performed in adults and there is only scarce knowledge on repertoire configurations in infants or children in general. Even more so, it is unclear if specific repertoire architectures in newborns, infants or children are informative for infection vulnerability. Longitudinally monitored cohorts with high-quality and high-resolution information about infectious episodes as well as a standardized sampling of biomaterials are required to study such questions.

Here, we used the unique LoewenKIDS cohort to study peripheral blood adaptive immune repertoires of 120 infants at the age of 12 months along with infection-related metadata^18^. This study provides the highest resolution data derived from daily symptom diaries in the first 6 years of life along with standardized questionnaires at defined time points, thereby allowing meaningful correlations of immune repertoire metrics with susceptibility to acute respiratory infections (ARI). The data presented here show an association between early life T cell repertoire restriction with respiratory tract infections. The database published along with this manuscript represents a valuable resource as repository for millions of immune receptor sequences from healthy infants with available metadata.

## Results

### Characteristics of the LoewenKIDS birth subcohort

**Figure 1:**
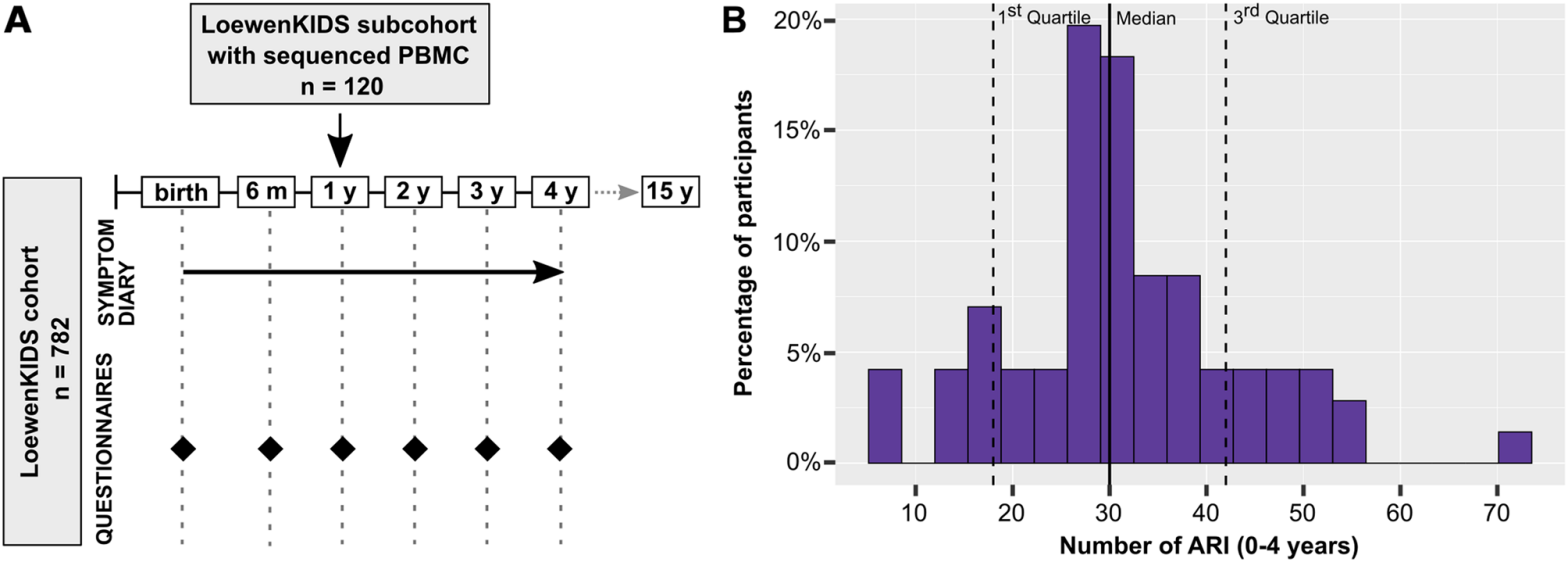
Acute respiratory infections (ARI) in the LoewenKIDS subcohort. (**A**) Visual description of the study design. (**B**) ARI episodes of all children with > 80% symptom diary completeness (n = 67) in the first 4 years of life.

The LoewenKIDS birth cohort recruited 782 newborns between November 2014 and February 2018 in five regions in Germany (Clinicaltrials.Gov Identifier: NCT02654210). A detailed description of the study design is provided elsewhere^18^. Briefly, participants were recruited prior to birth or until the age of 3 months and are being followed up until the age of 15 years. Parents fill in questionnaires at birth, at 6 months, at 12 months, and then annually until the age of 15 years (Fig. 1A). Questionnaires contain information on pregnancy and birth, social and health characteristics, as well as on diseases and environmental factors. Moreover, parents keep a daily symptom diary in the first 6 years of life. A subcohort of 120 cases donated blood at 12 months for immunological analyzes. This subcohort was used for the analyzes presented in this manuscript. Basic characteristics of this subcohort are given in Table 1, detailed metadata are included as Supplemental Table 1.

**Table 1:** Characteristics of LoewenKIDS subcohort.

	LoewenKIDS subcohort
**Number of participants**	120
**Sex**	
Female	57
Male	62
n.a	1
**Birth mode**	
Vaginal	92
C-section	28
**Older siblings**	
No	73
Yes	47
**Pets**	
No	77
Yes	43
**Onset of daycare**	
< 12 months	18
≥ 12 months	74
n.a	28
**Exclusive breastfeeding**	
No exclusive breastfeeding	3
< 6 months	49
≥ 6 months	51
n.a	17
**Total breastfeeding**	
No breastfeeding	0
< 6 months	13
6–12 months	44
≥ 12 months	52
n.a	11

n.a., not available.

All except one child were born within term (4 weeks prior to two weeks after the calculated date of birth).

### Acute respiratory infections (ARI) in the LoewenKIDS birth subcohort

Assessment of ARI was one major focus in this cohort study. ARI were classified based on daily symptom recordings throughout the first 6 years of life as described in the methods section. The youngest children in this subcohort completed their fourth year of life. The median numbers of ARI from birth to 1, 2, 3, and 4 years of age in this subcohort were 7 (interquartile range, IQR 5; 9), 18 (IQR 14; 21), 25 (IQR 19; 29), and 30 (IQR 26; 38), respectively. The distribution of the number of ARI among study participants in the first 4 years of life is shown in Fig. 1B.

### Global T and B cell immune metrics in infants from the LoewenKIDS birth subcohort versus a control study population of older individuals

We determined the peripheral blood immune repertoire architecture of T and B cells in our cohort of 120 infants by next-generation sequencing of the T cell receptor beta chain (TRB) and immunoglobulin heavy chain (IGH) locus. As part of physiological immune aging, immune repertoire restriction increases over the lifespan^16,17,19–21^. This process is reflected by a gradual increase in peripheral blood immune repertoire clonality and by loss of richness and diversity. To measure immune repertoire features of infancy in our cohort and to be able to perform meaningful comparisons, we included a total of 711 immune repertoire analyzes from older individuals sampled from the 1st to the 9th decade of life as a reference study population. Age distributions of these cases are shown in Supplemental Table 2.

**Figure 2:**
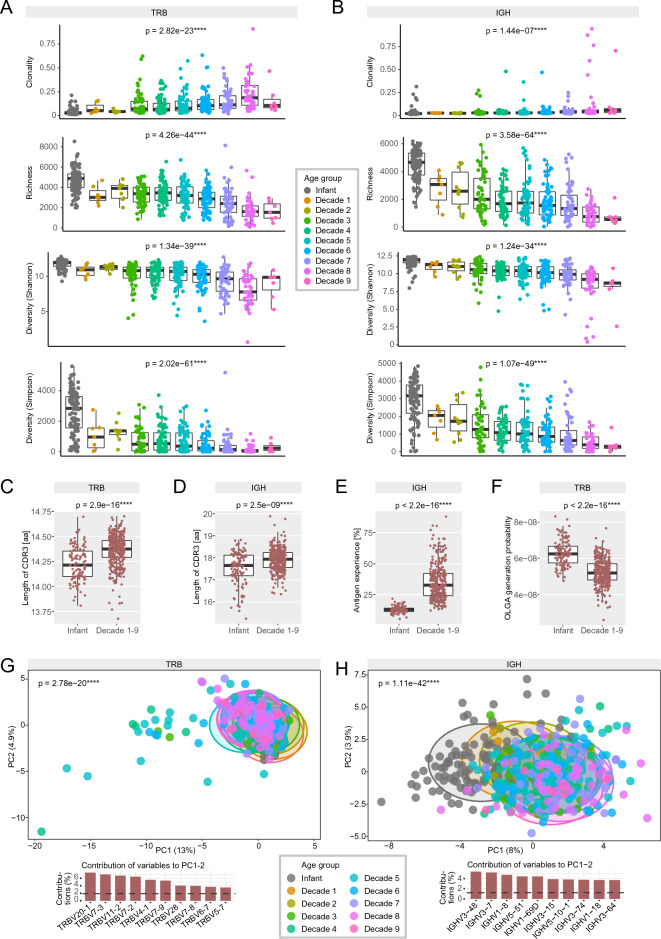
Blood immune repertoire metrics of LoewenKIDS subcohort sampled at 12 months compared to older control individuals sampled in their 1st to 9th decade of life. (**A**) T cell receptor (TCR) repertoire clonality, richness and two diversity measures are shown for the LoewenKIDS subcohort (“infant”) versus control immune repertoires from older individuals in their 1st to 9th decade of life (“decade 1–9”; dec). n(dec0) = 116, n(dec1) = 6, n(dec2) = 9, n(dec3) = 54, n(dec4) = 69, n(dec5) = 70, n(dec6) = 63, n(dec7) = 54, n(dec8) = 43, n(dec9) = 7. (**B**) B cell receptor (BCR) repertoire clonality, richness and two diversity measures are shown for the LoewenKIDS subcohort (“infant”) versus control immune repertoires from older individuals in their 1st to 9th decade of life (“decade 1–9”; dec). n(dec0) = 116, n(dec1) = 7, n(dec2) = 11, n(dec3) = 49, n(dec4) = 55, n(dec5) = 61, n(dec6) = 60, n(dec7) = 48, n(dec8) = 38, n(dec9) = 7. (**C**) Mean lengths of TCR complementarity-determining region 3 (CDR3) in LoewenKIDS subcohort (“infant”; n = 116) versus controls (“decade 1–9”; n = 377). (**D**) Mean lengths of BCR CDR3 in LoewenKIDS subcohort (“infant”; n = 116) versus controls (“decade 1–9”; n = 336). (**E**) Somatic hypermutation (SHM) of BCR in LoewenKIDS subcohort (“infant”) versus controls (“decade 1–9”). (**F**) Generation probability (Pgen) of TCR rearrangements in LoewenKIDS subcohort (“infant”) versus controls (“decade 1–9”). (**G**) Principal component analysis (PCA) of TCR V gene usage in LoewenKIDS subcohort (“infant”) versus controls in their 1st to 9th decade of life. V genes contributing most to the repertoire skewing across all age groups are shown. The dotted line indicates the contribution if variables were evenly distributed. (**H**) PCA of BCR V gene usage in LoewenKIDS subcohort (“infant”) versus controls in their 1st to 9th decade of life. V genes contributing most to the repertoire skewing across all age groups are shown. The dotted line indicates the contribution if variables were evenly distributed. One-way ANOVA was used for Panels (**A**) and (**B**). For Panels (**C**–**F**) an unpaired two-tailed t-test was performed. For Panels G and H, Pillai-MANOVA was used as statistical test. Analyses and data plotting were performed using RStudio (version 1.1.456) and the tcR, ade4 and tidyverse packages.

Overall, > 6 million T and > 10 million B cell receptor sequences were acquired from the LoewenKIDS infants. These were compared to > 44 million antigen receptor sequences from the control individuals. In line with prior data on immune aging^16^, infants from the LoewenKIDS cohort showed substantially lower T and B cell clonality as well as higher richness and diversity at the age of 12 months as compared to older individuals sampled in their 1st to 9th decade of life (Fig. 2A and B). The mean length of the T cell receptor complementarity-determining region 3 (CDR3) was 14.2 amino acids, that of the B cell receptor 17.5. Both were shorter than the respective CDR3 of older individuals which ranged around 14.4 for T and 17.9 for B cells consistent with the finding of shorter CDR3 in fetal lymphocytes^7,22^ (Fig. 2C and D). The rate of somatic hypermutation of B cell receptors was substantially lower than that of older individuals reflecting fewer episodes of antigenic challenge (Fig. 2E).

Next, we assessed the level of shared antigen receptor sequences in infants. Antigen receptors are generated stochastically in a multi-step process of genetic recombination. We calculated the probability of generation for each individual T cell receptor sequence (generation probability, Pgen) using the OLGA algorithm^23^. Sequences with high Pgen (> 1/10^9^) are frequently shared between individuals and are called public clonotypes, while private clonotypes have a low Pgen and are more infrequently shared by different individuals^24–29^. In our study populations, the calculated mean generation probability of all T cell rearrangements per repertoire decreased with increasing age (Fig. 2F). This suggested the accumulation of private T cell receptor clonotypes over the life span. More surprisingly however, the distribution of V genes in T and B cell receptor repertoires showed progressive age-dependent shifts (Fig. 2G and H). Especially IGHV families IGHV3-48, IGHV3-7 and IGHV1-8 showed a skewed distribution over the life span.

Together, low antigen-dependent somatic hypermutation of B cell repertoires as well as short CDR3 sequences, low T and B cell repertoire clonality, high diversity and high richness especially in public T cell clonotypes spoke in favor of a certain immunological naivety with high thymic and bone marrow output which is commonly observed at this developmental stage^30–32^ and relatively low numbers of antigen encounters until the sampling time point.

### Association of immune repertoire metrics with the number of ARI in the first 4 years of life

**Figure 3:**
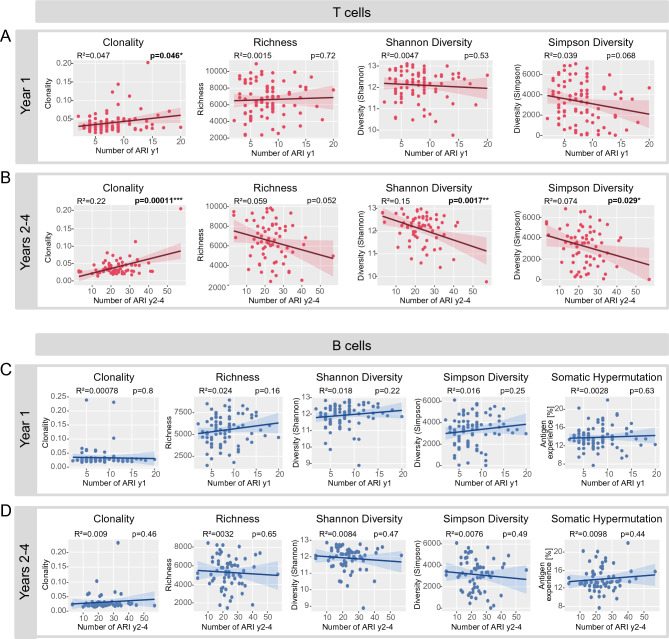
Correlation of blood immune cell metrics with acute respiratory infections (ARI) in LoewenKIDS subcohort. (**A**) T cell receptor (TCR) repertoire clonality, richness and two diversity measures are shown for the LoewenKIDS subcohort in relation to ARI in the 1st year of life. (**B**) TCR repertoire clonality, richness and two diversity measures are shown for the LoewenKIDS subcohort in relation to ARI in the 2nd to 4th year of life. (**C**) B cell receptor (BCR) repertoire clonality, richness, two diversity measures and somatic hypermutation (SHM) are shown for the LoewenKIDS subcohort in relation to ARI in the 1st year of life. (**D**) BCR repertoire clonality, richness, two diversity measures and SHM are shown for the LoewenKIDS subcohort in relation to ARI in the 2nd to 4th year of life. Only subjects with > 80% of days covered in the symptom diary were included in the analyses (89 subjects for the analysis of year 1 and 65 subjects for the analysis of clonality as well as 66 subjects for the analysis of the other immune metrics in year 2–4). One-way ANOVA was used as statistical test and squared Pearson correlation coefficients R^2^ are shown. Analyses and data plotting were performed using RStudio (version 1.1.456) and the tcR, ade4 and tidyverse packages.

**Figure 4:**
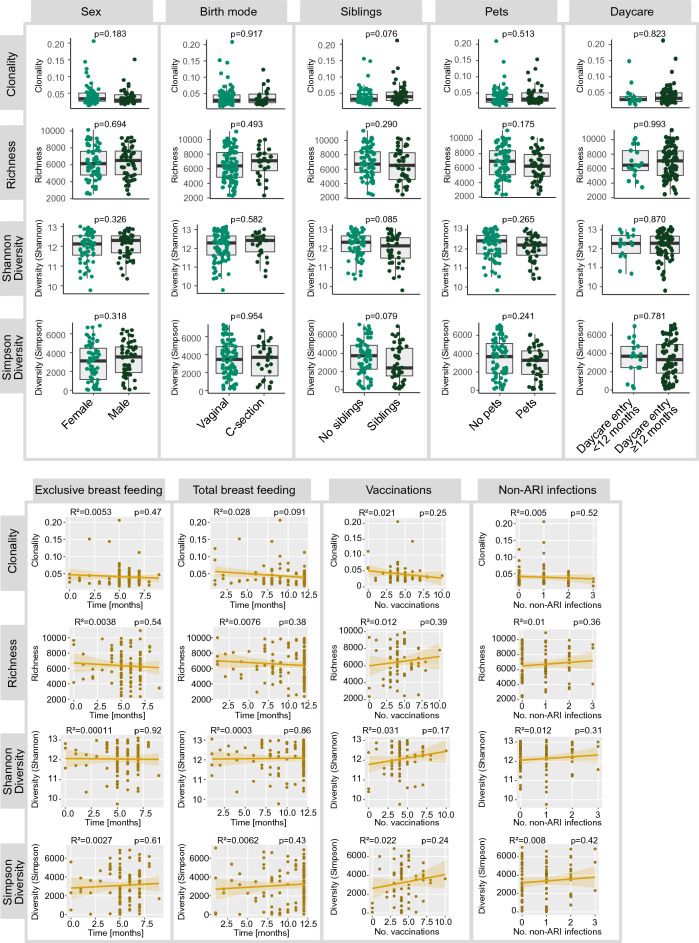
Correlation of blood T cell metrics with birth mode, breast feeding, siblings, daycare and other potential determinants in LoewenKIDS subcohort. T cell receptor (TCR) repertoire clonality, richness and two diversity measures are shown for the LoewenKIDS subcohort in relation to sex, birth mode, siblings, pets, onset of daycare and duration of breast feeding. Unpaired two-tailed t-test and one-way ANOVA were used as statistical tests. Squared Pearson correlation coefficients R^2^ are shown. Analyses and data plotting were performed using RStudio (version 1.1.456) and the tcR, ade4 and tidyverse packages.

Next, we correlated the reported number of ARI with immune repertoire metrics at 12 months of age. We observed that infants with restricted T cell receptor repertoires at 12 months of age—high clonality or low diversity—showed an overall higher vulnerability to ARI (Fig. 3A and B). This association was evident for the numbers of ARI in the 1st year of life (prior to blood sampling) and for ARI in the 2nd to 4th year of life (after blood sampling). No association was found between B cell repertoire metrics or somatic hypermutation and ARI (Fig. 3C and D). However, males showed lower levels of somatic hypermutation than females (Supplemental Fig. 1). Notably, we also observed slight associations of B cell repertoire diversity with the number of vaccinations and non-ARI infections (Supplemental Fig. 1). No other associations of immune cell metrics with any of the parameters assessed in the LoewenKIDS subcohort were found. Specifically, no correlation of immune cell metrics with birth mode, older siblings, pets, onset of daycare, duration of breast feeding, numbers of vaccinations or non-ARI infections were seen (Fig. 4 and Supplemental Fig. 1). We also did not observe any association of microbiome diversity with B or T repertoire metrics (Supplemental Fig. 2).

For a better interpretation of the association of immune metrics with the number of ARI, especially in years 2–4, we compared these with the effect of older siblings on the number of ARI, since a previous analysis of the overall LoewenKIDS cohort had demonstrated that older siblings were associated with more ARI in the first 2 years of life^33^. Given the correlations among the different immune repertoire indices, we studied each of them separately, always including the older sibling effect in the model.

**Figure 5:**
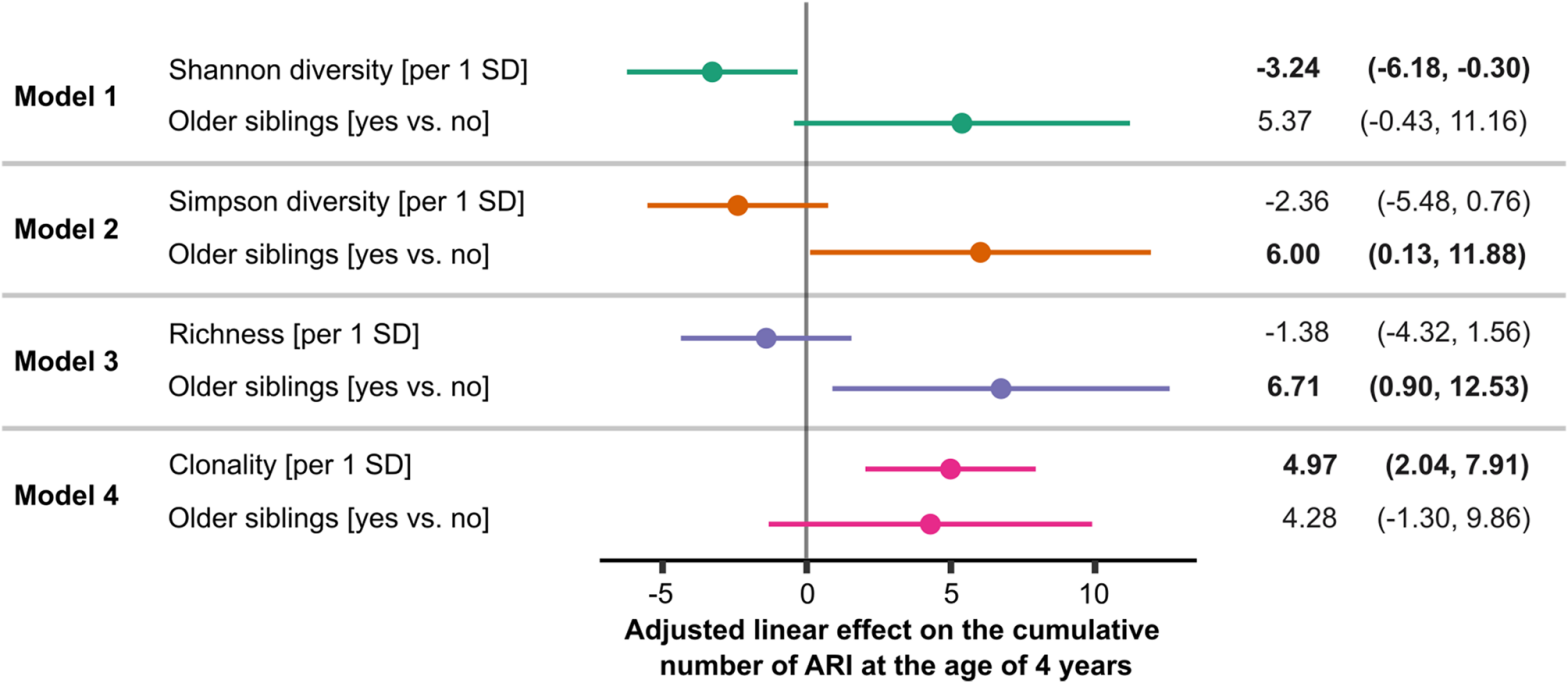
Adjusted linear effect on the cumulative number of ARI at the age of 4 years. Separate models show the association for each T cell repertoire index (Shannon diversity, Simpson diversity, richness, clonality) in comparison with the effect of older siblings, while mutually adjusting (n = 67). Estimates and 95% confidence intervals are shown. Estimates indicate number of additional infections. Analyses and data plotting were performed using RStudio (version 1.1.456).

Among the 67 children included in the analysis, the mean cumulative number of ARI in the first 4 years of life per participant was 31. Children with older siblings showed 4–7 additional episodes of infection in comparison to children without older siblings (Fig. 5). The effects of higher clonality and lower Shannon diversity (per 1 standard deviation, SD) were only slightly smaller, 5 and 3 additional infections in the first 4 years, respectively, followed by lower Simpsons` diversity and lower richness (Fig. 5).

Whereas the effects of immune metrics on the number or ARI gradually increased within the first 4 years of life, the effect of older siblings decreased with age (Supplemental Fig. 3).

## Discussion

In this study, we found high immunological richness and diversity along with signs of immunological naivety in the majority of T and B cell repertoires of 12 months old infants compared to a large cohort of individuals of all ages. Infants with age-inadequate T but not B cell repertoire restriction showed higher numbers of respiratory infections in the first 4 years of life. This—to our knowledge—is the first human study demonstrating a link between T cell repertoire metrics and immunity to infection early in life. It supports that a preexistent variety of T cell receptors is a fundamental prerequisite for immunological recognition of the universe of foreign antigens and that, consequently, T cell immune metrics may represent a predictor for infection susceptibility in infants. While T cell receptor repertoire diversity may not fully mirror functional competence, this data underlines the importance of repertoire breadth in immunity to infection as suggested by a couple of previous studies on immune protection^34–36^. More indirectly, this paradigm is also supported by emerging data on blood T cell receptor repertoire diversity and richness as biomarkers for response to cancer immunotherapy^37–39^.

While immune repertoire restriction likely causes infection vulnerability, the study design cannot rule out that the observed restriction in some infants may also be the consequence of previous infections. The fact that blood sampling in this study was conducted at 12 months of age and no blood samples from an earlier time point were available, therefore, represents one of the limitations of this study. Yet, it needs to be noted that the expansion of antigen-specific (e.g., CD8+) T cell clones in secondary lymphoid organs in response to infectious triggers generally does not translate into a more clonal blood T cell repertoire, as shown for acute COVID-19^40^. In contrast, a reactive increase in blood T cell receptor repertoire richness and drop in clonality below the steady state can be observed in the weeks following the acute phase of antigenic challenge^40^. In this line of reasoning, children with high infection burden should rather show low repertoire clonality and reactively increased richness.

Potential determinants of T cell repertoire restriction in these infants—beyond presumable genetic predisposition—remain largely unclear. None of the basic parameters investigated in the LoewenKIDS cohort appeared to show correlation with T cell metrics. Most importantly, no association of the birth mode or the duration of breast-feeding was found. This is of interest since the composition of immune cells seems to be affected by these parameters in newborns^41–44^. Furthermore, the maturation of adaptive immunity is influenced by bacterial colonization immediately after birth^1^. A number of prior studies have suggested effects of caesarean sections and breast-feeding on microbiome shaping, but there is limited evidence on potential microbiome-mediated effects on infection vulnerability^45–49^. Future studies explicitly addressing potential effects of the microbiome on T cell repertoire shaping are certainly warranted.

With a total of 943 immune repertoires, this study provides a unique resource for researchers in the field. These repertoires contain > 60 million antigen receptor sequences from individuals across all age and sex groups. The drastic drop in repertoire diversity and mutation status already within the first decade of life emphasize the rapid dynamics of the adaptive immune system in early infancy. This is in line with longitudinal studies on soluble factors and cell composition in these first important years^50–52^. The absolute number of lymphocytes peaks during the first months after birth followed by a steady decrease^8,50,53^, which likely contributes to the higher repertoire richness observed in the LoewenKIDS infants. Another notable observation was the reduced level of somatic hypermutation in males of the LoewenKIDS cohort. Sex has long been recognized as functional variable in immunity^54,55^. Females, for example, tend to mount stronger immune responses to viral and bacterial infections or vaccinations but develop autoimmunity more often^54,55^. Interestingly, a similar sex-related pattern of somatic hypermutation was reported in older individuals and linked to differential expression of DNA repair genes^56^. A presumable driver of this pattern are sex-related steroid hormones like estradiol, which exhibit specific post-natal activation patterns and can induce affinity maturation and class switch recombination^54,57^. Another important finding in this large comparative analysis is the progressive shift of immune repertoires towards antigen receptors with specific V gene usage. Similar findings of progressive age-related skewing of B cell repertoires have been described in previous studies^52,58^, but—expectedly—could not be detected in a study with only 20 immune repertoires^59^. Our study, thereby, confirms prior evidence that repertoire diversity progressively declines during life and that repertoire depletion does not uniformly affect all clonotypes to the same extent. It might seem counterintuitive at first glance that adult CDR3 are longer than those of infants although infant repertoires contain a significantly higher number of naïve B cell receptor sequences which are on average longer than mutant ones. Yet, this has been observed before in smaller cohorts^7,60–63^. This finding might be explained by the absence of terminal deoxynucleotidyl transferase (TdT) expression before the third trimester, which results in a lack of random nontemplated (N) nucleotide insertions to rearranged CDR3 regions^7,63–65^ or by the fact that B cell receptor rearrangements with long CDR3 sequences are more prone to mediate autoimmunity and are therefore preferentially removed during cell maturation^66^. This again links younger age with higher immune fitness and an accumulation of autoimmune events at increasing age. These insight into repertoire dynamics over life may foster a better understanding of immunosenescence in healthy aging, immunosurveillance in cancer and emergence of autoimmunity. It should also raise our awareness of using age-matched control cohorts in immunosequencing studies in general.

## Conclusions

Together, this study shows that the majority of infants at 12 months of life have highly rich and diverse immune repertoires with low clonality that show only limited imprints of previous antigenic challenge. Our results also support the notion that an age-adequate diversity and clonality of the T cell space is associated with lower numbers of acute respiratory infections in the first 4 years of life.

## Methods

### Study design and biobanking of the LoewenKIDS subcohort and control cohort

LoewenKIDS is a cohort study conducted in five cities in Germany (Braunschweig, Hannover, Halle, Munich, Bremen) which registered 782 participants until February 2018^18^. The recruitment was conducted via antenatal preparation courses, information evenings in hospitals and private practices. The study was approved by the Ethics Committees of the Martin-Luther-University Halle-Wittenberg (protocol code 2016-04 from 20 April 2016), Hannover Medical School (protocol code 6794 from 11 November 2014) and Ludwig-Maximilians-University Munich (protocol code 445-15 from 24 September 2015) and conducted in accordance with the ethical principles stated by the Declaration of Helsinki. Informed written consent was obtained from all participants or legal representatives. 120 infants enrolled on this cohort study with available blood samples from the 12 months time point were included in this subcohort analysis.

Metadata was derived from questionnaires provided to participants at 6 months, as well as at one, two, three, and 4 years of life and contained information on pregnancy and birth, social and health characteristics, as well as on diseases and environmental factors. Entries from the first 4 years of life of the daily symptom diary were used for this analysis. In the symptom diary, parents record A-symptoms (fever, wheezing, wet cough, and medically-diagnosed pneumonia or otitis media) and B-symptoms (dry cough, chills, sore throat, runny or blocked nose, increased need to sleep, loss of appetite, and increased attachment). ARI were classified based on A- and B-symptoms where at least one A-symptom or a day with two B-symptoms was sufficient to define the beginning of an ARI episode^18,33^.

As a control, a total of 711 blood T and B cell receptor repertoire analyzes from individuals above 1 year of age (sampling in 1st to 9th decade of life) were used. A history of cancer was an exclusion criterion for this control cohort, since inadequately high peripheral blood immune repertoire restriction is common in cancer patients. Informed written consent was obtained as approved by the ethics commission Hamburg (Ethikkommission der Ärztekammer Hamburg, Germany, project number PV4767) the ethics commission Halle (Project No. 2014-75 and 2020-076) and by the National Human Genome Research Institute’s Institutional Review Board. A subset of the control sequencing analyzes have been previously published^16,17^.

### Sample collection

Peripheral mononuclear cells (PBMC) were isolated from blood by standard Ficoll gradient centrifugation. Genomic DNA was extracted from PBMCs using the GenElute Mammalian Genomic DNA Miniprep Kit (Sigma-Aldrich, St. Louis, USA). Fecal samples were collected according to institutional practices and frozen at − 80 °C until RNA extractiont.

### Next-generation sequencing of T and B cell immune repertoires

V(D)J rearranged immunoglobulin heavy (IGH) and T cell receptor beta (TRB) loci were amplified from 250 ng of genomic DNA using a multiplex PCR and the BIOMED2-FR1 (*IGH*) or—*TRB* primer pools^67^, pooled at 4 nM and quality-assessed on a 2100 Bioanalyzer (Agilent Technologies). Sequencing was performed on an Illumina MiSeq (paired-end, 2 × 301-cycles, v3 chemistry). Analysis of the rearranged IGH and TRB loci was computed using the MiXCR framework^68^. As reference for sequence alignment the default MiXCR library was used for TRB sequences and the IMGT library v3 for IGH. Each unique complementarity-determining region 3 (CDR3) nucleotide sequence was considered a clone. Non-productive reads and sequences with less than 2 read counts were not considered for further bioinformatics evaluation. All repertoires were normalized to the same read count. For analyses within the LoewenKIDS cohort repertoires were normalized to 50,000 reads. For comparison with control samples of other age groups repertoires were sampled to 30,000 reads due to a lower read depth of control samples. IGHV genes were regarded as somatically hypermutated if they showed < 98% identity to the germline sequence. All analyses and data plotting were performed using RStudio (version 1.1.456) and the tcR, ade4 and tidyverse packages.

### Immune repertoire metrics

We calculated the clonality of the sequenced IGH and TRB repertoires according to the formula “1-Pielou's evenness”. In our setting, evenness measures the relative abundance of unique B or T cell clones in the repertoire and is calculated according to the formula J = H'/log2(S) with H' being the Shannon diversity index and S the total clone number (richness) in a distinct sample. A clonality index of 1 indicates that the analysed sample contains only one clone whereas 0 indicates complete clonal diversity. As a second diversity measure, the Simpson index^69^ was used which is disproportionately sensitive to the most common species/clonotypes in the repertoire.

Pgen of each TRB clonotype was calculated using the OLGA (Optimized Likelihood estimate of immunoGlobulin Amino-acid sequences) algorithm with default parameters^23^. The mean Pgen of all clonotypes per repertoire was plotted.

### Regression analysis

We used linear regression to determine the associations between standardized T cell repertoire measures (clonality, richness, Simpson diversity index, Shannon diversity index), the presence of older siblings, and the number of ARI until the age of 4 years in 67 children (56%) with ≥ 80% complete symptom diary data. In addition, we used Poisson regression to generate comparable estimates across various time intervals, the reported relative risks indicate relative increase / decrease in the number of cumulative infections per one SD of the standardized metrics and for children with or without older siblings. Effect estimates and their corresponding 95% confidence intervals (95% CI) are presented. All regressions were performed in R (Version 4.2.0).

### Stool specimen processing

16S rRNA sequencing of the V4 Region of gut microbiome was performed through Illumina MiSeq sequencing. Resulting data were demultiplexed, barcodes and primers removed.

### Microbiome data analysis

Read pairs were merged samplewise using USERACH (v11.0.667) were we set—fastq_maxdiffs 10 because of long overlaps. Average depth per file was 33 k read pairs. Reads were filtered (-fastq_maxee 1.0) and unique sequences were determined. Sequences were then clusterd into OTUs (operating taxonimic units) at a similarity of 97% using the UPARSE-OTU algirithm and denoised to zOTUs (zero-radius OTUs) with unoise3 algorithm [ref] implemented in USEARCH. Files with a read depth smaller than 9000 mapped reads were discarded. All samples were then subsampled to a read depths of 9000. Alpha diversity and other metrics were calculated, genus and phylum were assigned with sintax algorithm^70^ implemented in USEARCH to the 16 s RDP Database (v.18).

### Statistics

Boxplots are presented in the style of Tukey. *P*-values for comparison of two groups were calculated using unpaired two-tailed t-test. Data were tested for normality, variance and linearity (Supplemental Fig. 4) and thus, *P*-values for comparison of more than two groups were calculated using the parametric one-way ANOVA. In principal component analyses (PCA) Pillai-MANOVA was used as statistical test. The ellipse in PCA plots refers to three times the Euclidian distance. Pearson correlation (R) was used for linear regression fits, R^2^ and 95% confidence intervals are shown. Data for ARI were only used of those participants with > 80% complete symptom diary data (89 subjects for the analysis of year 1 and 66 subjects for the analysis of year 2–4). All statistical analyses were performed using R version 4.1.2.

### Ethics approval and consent to participate

The study was approved by the Ethics Committees of the Martin-Luther-University Halle-Wittenberg (protocol code 2016-04 from 20 April 2016), Hannover Medical School (protocol code 6794 from 11 November 2014) and Ludwig-Maximilians-University Munich (protocol code 445-15 from 24 September 2015) and conducted in accordance with the ethical principles stated by the Declaration of Helsinki. Informed written consent was obtained from all participants or legal representatives.

## Supplementary Information

Supplementary Figure 1.

Supplementary Figure 2.

Supplementary Figure 3.

Supplementary Figure 4.

Supplementary Table 1.

Supplementary Table 2.

## Data Availability

The herein reported sequence data set has been deposited at the European Nucleotide Archive (ENA) https://www.ebi.ac.uk/ena/browser/view/PRJEB58155.
