# Supplementary material for: T cell repertoire breadth is associated with the number of acute respiratory infections in the LoewenKIDS birth cohort
Source: Sci Rep. 2023 Jun 12;13:9516. doi: 10.1038/s41598-023-36144-x (PMC10258752; doi:10.1038/s41598-023-36144-x)
Supplement: Supplementary file 1 — Supplementary Figure 1. [file 41598_2023_36144_MOESM1_ESM.pdf]

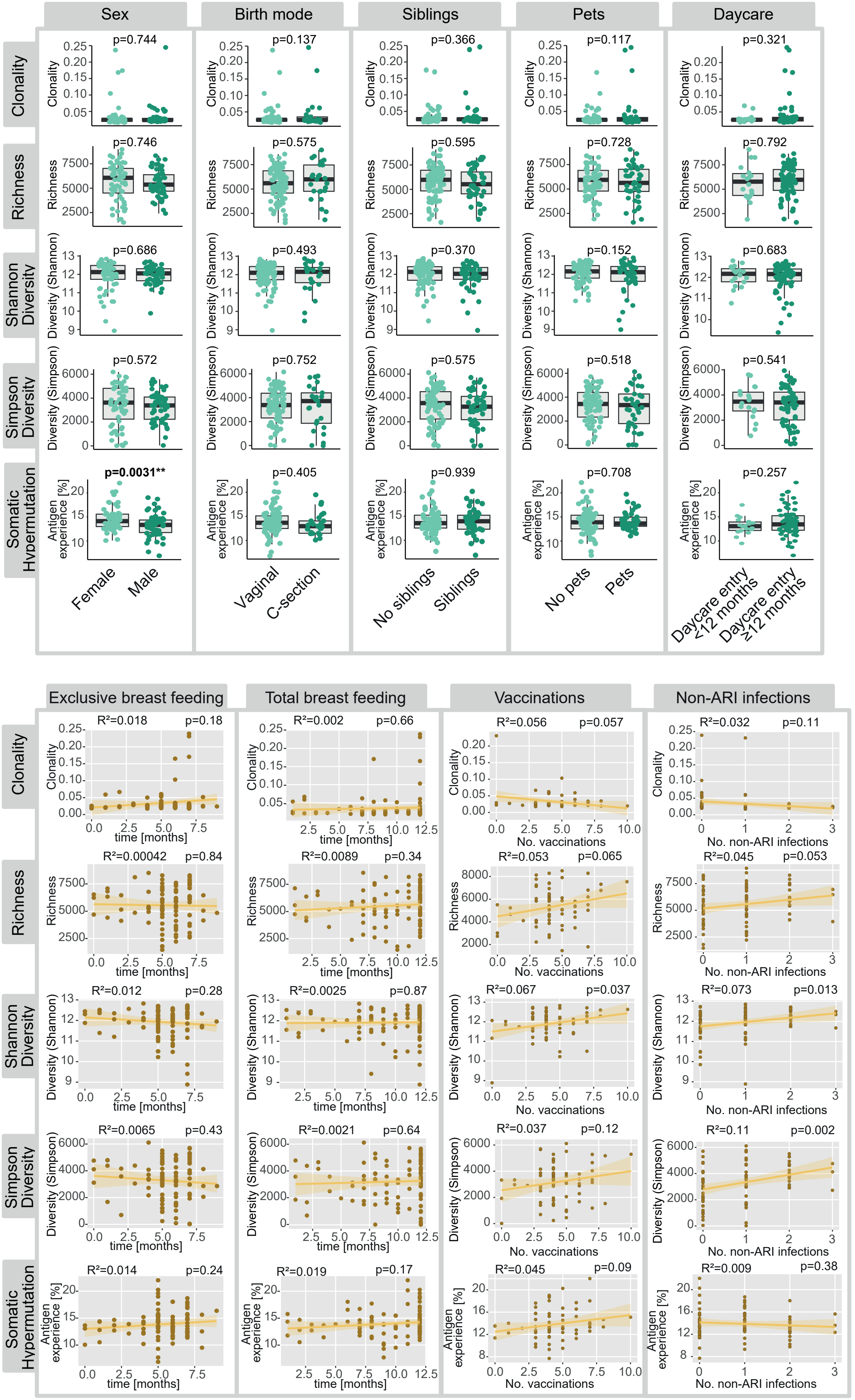


**Supplemental Figure 1:** Correlation of blood B cell metrics with birth mode, breast feeding, siblings, daycare and other potential determinants in LoewenKIDS subcohort. B cell receptor (BCR) repertoire clonality, richness, two diversity measures and somatic hypermutation are shown for the LoewenKIDS subcohort in relation to sex, birth mode, siblings, pets, onset of daycare and duration of breast feeding as well as numbers of vaccinations and non-ARI infections. Unpaired two-tailed t-test and one-way ANOVA were used as statistical test. Squared Pearson correlation coefficients $R^2$ are shown.
