# Supplementary material for: T cell repertoire breadth is associated with the number of acute respiratory infections in the LoewenKIDS birth cohort
Source: Sci Rep. 2023 Jun 12;13:9516. doi: 10.1038/s41598-023-36144-x (PMC10258752; doi:10.1038/s41598-023-36144-x)
Supplement: Supplementary file 2 — Supplementary Figure 2. [file 41598_2023_36144_MOESM2_ESM.pdf]

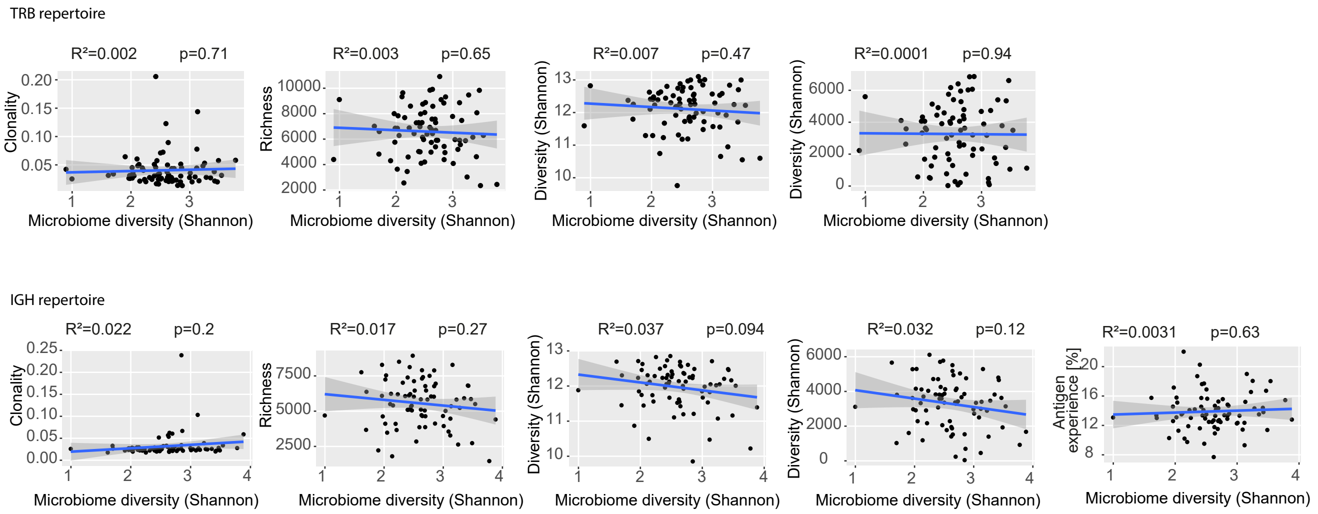

Supplemental Figure 2: Correlation of blood B cell metrics with microbiome diversity in LoewenKIDS subcohort. Correlations displayed for TRB and BCR repertoires. Unpaired two-tailed t-test and one-way ANOVA were used as statistical test. Squared Pearson correlation coefficients R^2 are shown.
