# Supplementary material for: T cell repertoire breadth is associated with the number of acute respiratory infections in the LoewenKIDS birth cohort
Source: Sci Rep. 2023 Jun 12;13:9516. doi: 10.1038/s41598-023-36144-x (PMC10258752; doi:10.1038/s41598-023-36144-x)
Supplement: Supplementary file 3 — Supplementary Figure 3. [file 41598_2023_36144_MOESM3_ESM.pdf]

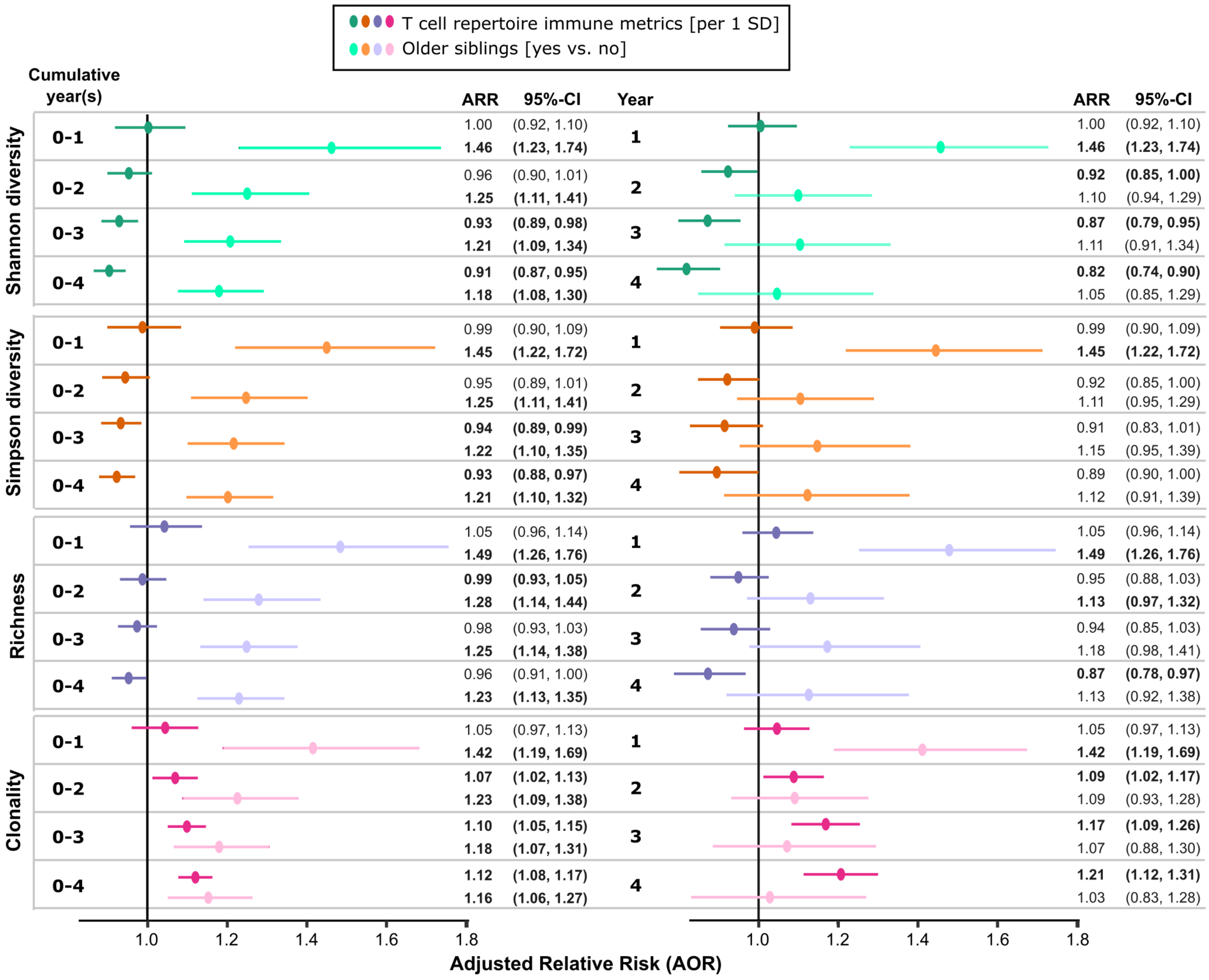

Metric	Cumulative year(s)	ARR	95%-CI	Year	ARR	95%-CI
Shannon diversity	0-1	1.00	(0.92, 1.10)	1	1.00	(0.92, 1.10)
		**1.46**	**(1.23, 1.74)**		**1.46**	**(1.23, 1.74)**
	0-2	0.96	(0.90, 1.01)	2	**0.92**	**(0.85, 1.00)**
		**1.25**	**(1.11, 1.41)**		1.10	(0.94, 1.29)
	0-3	**0.93**	**(0.89, 0.98)**	3	**0.87**	**(0.79, 0.95)**
		**1.21**	**(1.09, 1.34)**		1.11	(0.91, 1.34)
	0-4	**0.91**	**(0.87, 0.95)**	4	**0.82**	**(0.74, 0.90)**
		**1.18**	**(1.08, 1.30)**		1.05	(0.85, 1.29)
Simpson diversity	0-1	0.99	(0.90, 1.09)	1	0.99	(0.90, 1.09)
		**1.45**	**(1.22, 1.72)**		**1.45**	**(1.22, 1.72)**
	0-2	0.95	(0.89, 1.01)	2	0.92	(0.85, 1.00)
		**1.25**	**(1.11, 1.41)**		1.11	(0.95, 1.29)
	0-3	**0.94**	**(0.89, 0.99)**	3	0.91	(0.83, 1.01)
		**1.22**	**(1.10, 1.35)**		1.15	(0.95, 1.39)
	0-4	**0.93**	**(0.88, 0.97)**	4	0.89	(0.90, 1.00)
		**1.21**	**(1.10, 1.32)**		1.12	(0.91, 1.39)
Richness	0-1	1.05	(0.96, 1.14)	1	1.05	(0.96, 1.14)
		**1.49**	**(1.26, 1.76)**		**1.49**	**(1.26, 1.76)**
	0-2	**0.99**	**(0.93, 1.05)**	2	0.95	(0.88, 1.03)
		**1.28**	**(1.14, 1.44)**		**1.13**	**(0.97, 1.32)**
	0-3	0.98	(0.93, 1.03)	3	0.94	(0.85, 1.03)
		**1.25**	**(1.14, 1.38)**		1.18	(0.98, 1.41)
	0-4	0.96	(0.91, 1.00)	4	**0.87**	**(0.78, 0.97)**
		**1.23**	**(1.13, 1.35)**		1.13	(0.92, 1.38)
Clonality	0-1	1.05	(0.97, 1.13)	1	1.05	(0.97, 1.13)
		**1.42**	**(1.19, 1.69)**		**1.42**	**(1.19, 1.69)**
	0-2	**1.07**	**(1.02, 1.13)**	2	**1.09**	**(1.02, 1.17)**
		**1.23**	**(1.09, 1.38)**		1.09	(0.93, 1.28)
	0-3	**1.10**	**(1.05, 1.15)**	3	**1.17**	**(1.09, 1.26)**
		**1.18**	**(1.07, 1.31)**		1.07	(0.88, 1.30)
	0-4	**1.12**	**(1.08, 1.17)**	4	**1.21**	**(1.12, 1.31)**
		**1.16**	**(1.06, 1.27)**		1.03	(0.83, 1.28)

Supplemental Figure 3: Poisson regression analysis of the cumulative number of ARI at the age of 1, 2, 3, and 4 years and in the respective years alone. Separate models show the association for each T cell repertoire immune metric (Shannon diversity, Simpson diversity, richness, clonality) in comparison with the effect of having older siblings, while mutually adjusting within each time frame or year (n=67). Estimates and 95% confidence intervals are shown.
