# Supplementary material for: T cell repertoire breadth is associated with the number of acute respiratory infections in the LoewenKIDS birth cohort
Source: Sci Rep. 2023 Jun 12;13:9516. doi: 10.1038/s41598-023-36144-x (PMC10258752; doi:10.1038/s41598-023-36144-x)
Supplement: Supplementary file 4 — Supplementary Figure 4. [file 41598_2023_36144_MOESM4_ESM.pdf]

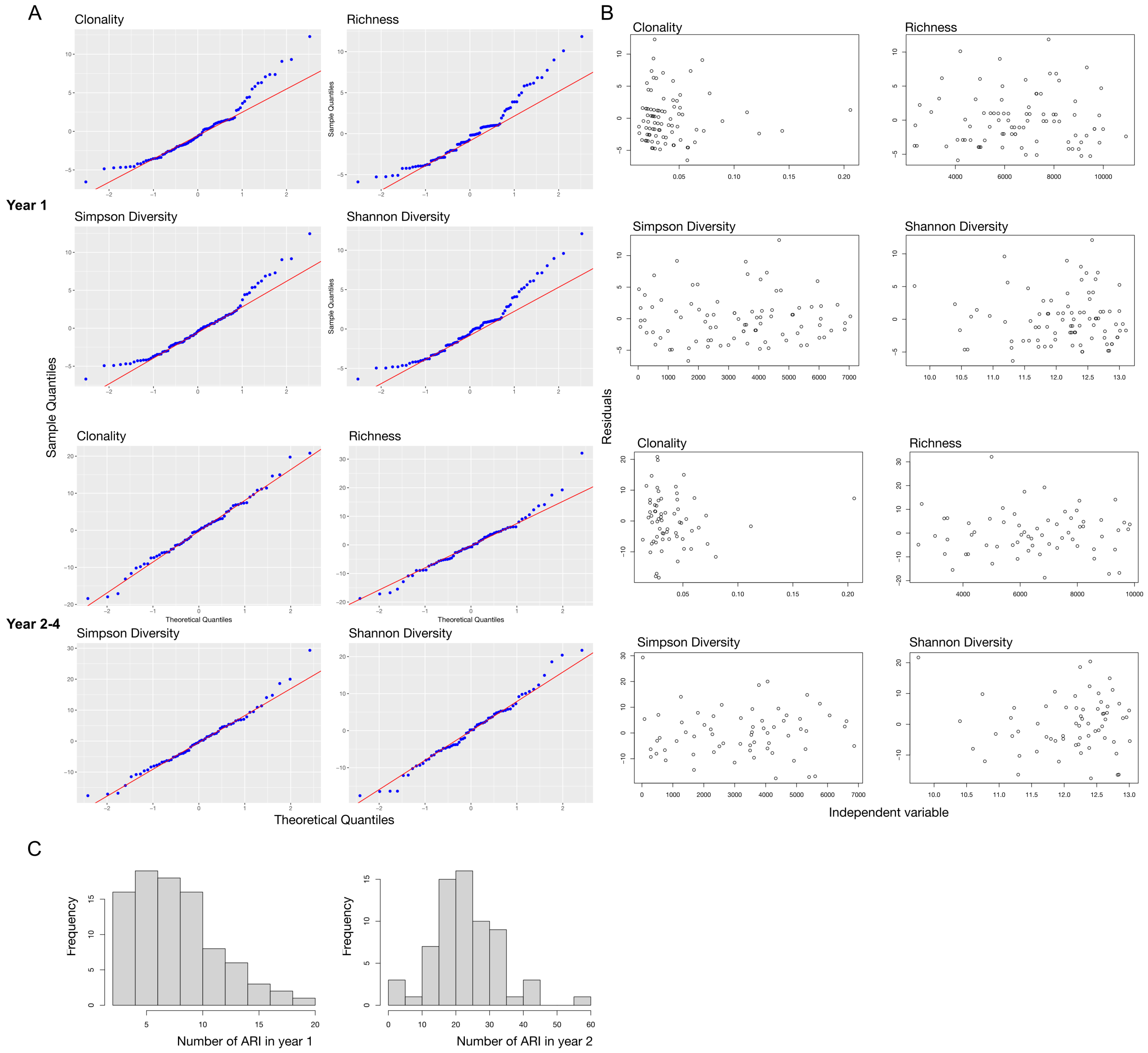

Supplemental Figure 4: Determination of statistical analysis method. (A) To test the similarity between the distributions of the number of ARI in year 1 as well as years 2-4 and respective immune metric measures, quantil-quantil-plots were used. (B) The independent variables (clonality, richness, Simpson diversity, Shannon diversity) were plotted against the respective modelled residuals to assess variance and linearity. (C) The frequency of the number of ARI in year 1 (n=89) and year 2-4 (n=66) of the LoewenKIDS participants with blood samples and symptom diary data completeness of >80% is shown.
